# Derivation of a novel multi‐gene prognostic model based on regulated cell death pathways in acute myeloid leukemia: A comprehensive bioinformatic analysis integrating gene expression, mutation profiling, and immune infiltration

**DOI:** 10.1371/journal.pone.0328412

**Published:** 2025-08-01

**Authors:** Ali Ahmadi, Amir Abas Navidinia, Davood Bashash, Behzad Poopak, Shadi Esmaeili

**Affiliations:** 1 Department of Hematology and Blood Banking, School of Allied Medical Sciences, Shahid Beheshti University of Medical Sciences, Tehran, Iran; 2 Hematology-Oncology and Stem Cell Transplantation Research Center, Tehran University of Medical Sciences, Tehran, Iran; 3 Department of Medical Laboratory Sciences, Faculty of Paramedical Sciences, Islamic Azad University, Tehran, Iran; European Institute of Oncology, ITALY

## Abstract

**Background:**

Acute myeloid leukemia (AML) is a highly aggressive hematologic malignancy with dismal survival outcomes, where dysregulation of regulated cell death (RCD) pathways plays a pivotal role in leukemogenesis and therapeutic resistance.

**Methods:**

Differential expression analyses were performed comparing AML samples with healthy bone marrow. Diagnostic differentially expressed genes (DEGs) were then intersected with curated gene sets representing apoptosis, pyroptosis, autophagy, necroptosis, and ferroptosis to derive an RCD-based gene signature. Prognostic markers were identified by univariate Cox regression, and these markers were refined using LASSO regression to construct a multi-gene prognostic model that generated an individual risk score (RS) for each patient. The performance of the model was validated internally through Kaplan–Meier survival analyses and receiver operating characteristic (ROC) curves for 1-, 3-, and 5-year survival, and externally confirmed in an independent TARGET-AML cohort. In addition, mutation analysis was conducted using the maftools package, and immune infiltration profiling was performed with CIBERSORT and xCell to characterize the molecular landscape of the risk groups.

**Results:**

Our integrative approach yielded a four-gene prognostic model incorporating ARHGEF35, GSN, ELANE, and AKT3. High RS was strongly associated with adverse overall survival, with Kaplan–Meier analyses showing *p-value* < 0.0001 in the training cohort and *p-value* = 0.0026 in the testing cohort. The model demonstrated robust predictive accuracy with AUC values of 82%, 87%, and 91% for 1-, 3-, and 5-year survival in the training set, and 65%, 81%, and 94% in the testing set. Mutation analysis revealed that DNMT3A and RUNX1 mutations were significantly enriched in high-RS patients (*p-value* = 0.0015 and *p-value* = 0.0086, respectively), whereas KIT mutations were more prevalent in low-RS patients (*p-value* = 0.0058). Immune profiling indicated that high-RS patients had increased M2 macrophage infiltration (*p-value* = 0.0027) and reduced resting mast cells (*p-value* = 0.0033).

**Conclusion:**

These findings establish that an RCD-based multi-gene risk model can robustly stratify AML patients by prognosis and illuminate underlying genomic and immunologic mechanisms, thereby offering promising avenues for personalized therapeutic strategies.

## 1 Introduction

Acute myeloid leukemia (AML) is a highly aggressive hematologic malignancy characterized by the clonal expansion of immature myeloid progenitors that supplant normal hematopoietic cells within the bone marrow, leading to severe cytopenias and profound immunosuppression [1]. The disease’s pathogenesis is driven by a complex interplay of genetic mutations, epigenetic modifications, and aberrant signaling cascades that not only foster malignant transformation but also contribute to the survival and proliferation of leukemic cells [2]. Despite significant advances in induction chemotherapy, relapse rates in AML remain unacceptably high, underscoring the urgent need for refined prognostic biomarkers and novel therapeutic strategies [2–5]. The heterogeneous nature of AML, molded by a mosaic of genetic, epigenetic, and microenvironmental factors, complicates clinical management and necessitates an integrated understanding of the cellular processes governing leukemic cell fate [6].

Regulated cell death (RCD) pathways, including apoptosis, pyroptosis, autophagy, necroptosis, and ferroptosis, have emerged as critical determinants of cell fate in AML [7]. Apoptosis, the most thoroughly characterized form of RCD, is orchestrated through intrinsic and extrinsic signaling cascades with the BCL-2 family of proteins serving as key modulators; disruptions in these pathways, such as the overexpression of anti-apoptotic proteins (e.g., BCL-2, MCL-1) and the suppression of pro-apoptotic effectors (e.g., BAX, BAK), have been directly linked to leukemogenesis and the emergence of chemoresistance [8,9]. In parallel, pyroptosis—an inflammasome-driven process marked by gasdermin-mediated membrane pore formation and the release of pro-inflammatory cytokines—appears to influence anti-tumor immunity and may offer novel immunomodulatory targets [10,11]. Furthermore, autophagy exhibits a dual role in AML by acting either as a cytoprotective mechanism under metabolic stress or as a trigger for autophagic cell death when dysregulated, emphasizing the delicate balance that governs cell survival and death [12,13]. Necroptosis provides an alternative cell death pathway in scenarios where apoptosis is impaired, while ferroptosis, driven by iron-dependent lipid peroxidation under conditions of heightened oxidative stress, represents an emerging therapeutic target in AML [11,14].

However, despite these advances, a comprehensive understanding of how these diverse RCD modalities integrate with genetic and epigenetic alterations to influence AML progression and therapeutic response remains elusive. The heterogeneous expression of RCD regulators across AML subtypes, and their intricate interplay with other cellular processes, highlights a significant gap in our current knowledge that hampers the development of predictive prognostic models and targeted therapies. In response to these challenges, our study introduces a novel prognostic model that integrates gene expression signatures associated with key RCD pathways.

## 2 Materials and methods

### 2.1 Data acquisition and preprocessing

Uniformly processed RNA-Seq data were obtained using the R package recount3 (version 1.14.0) from two discovery cohorts: (i) bone marrow samples from healthy individuals provided by the Genotype-Tissue Expression (GTEx) Project and (ii) AML samples from The Cancer Genome Atlas (TCGA). In addition, level 3 RNA sequencing data along with comprehensive clinical annotations for 151 newly diagnosed AML patients from TCGA and 312 AML patients from the Therapeutically Applicable Research to Generate Effective Treatments (TARGET) database were retrieved via the Genomic Data Commons (GDC) repository (https://portal.gdc.cancer.gov/repository). All datasets underwent rigorous quality control and normalization procedures to ensure consistency and facilitate subsequent integrative analyses.

### 2.2 Differential expression analysis and visualization

Differentially expressed genes (DEGs) between healthy bone marrow and AML samples were identified using the DESeq2 package. A Benjamini–Hochberg procedure was applied to control the false discovery rate, and genes with an adjusted p-value (FDR) below 0.05 and an absolute log₂ fold-change of at least 1 were considered significantly differentially expressed. Principal component analysis (PCA) was performed to assess the overall separation between the two groups, and a volcano plot was generated with the EnhancedVolcano package to provide a visual summary of the DEG distribution. Protein-coding DEGs meeting these criteria were designated as diagnostic-DEGs for further analyses.

### 2.3 Gene set annotation

To capture the contribution of RCD modalities, a comprehensive set of genes was annotated by retrieving gene sets from the REACTOME database using the msigdbr package. Gene sets associated with terms such as cell_death, apoptosis, pyroptosis, autophagy, necroptosis, and ferroptosis were identified, and the union of these gene sets was compiled to form a master list of RCD-related genes.

### 2.4 Prognostic gene selection

Diagnostic-DEGs were intersected with the RCD-related gene list to obtain a subset of apoptosis-related diagnostic DEGs. From the TCGA cohort, patients with incomplete clinical data, including missing survival information, were excluded. A univariate Cox regression analysis was then performed to identify genes significantly associated with overall survival. Genes demonstrating a p-value below 0.05 were classified as prognostic and apoptosis-related diagnostic DEGs (PDA-DEGs).

### 2.5 Construction and evaluation of the prognostic model

For model construction, the TCGA-LAML dataset was randomly partitioned into a training subset (60%) and a testing subset (40%), with the training set used to develop the model and the testing set (in addition to the entire TCGA dataset) used for internal validation. To reduce the risk of overfitting and select the most informative prognostic features, a least absolute shrinkage and selection operator (LASSO) regression was performed using the glmnet package with 10-fold cross-validation. The selected features were then incorporated into a multivariate Cox regression model. The risk score (RS) for each patient was calculated using the following gold-standard formula: RS = (expression of mRNA_1_ × coefficient_mRNA1_) + (expression of mRNA_2_ × coefficient_mRNA2_) + … + (expression of mRNA_n_ × coefficient_mRNAn_).

Model evaluation included generating forest plots to illustrate hazard ratios, as well as receiver operating characteristic (ROC) curves for 1-, 3-, and 5-year survival. The area under the curve (AUC) was computed for the TARGET cohort to assess predictive performance. Furthermore, correlations between RS and clinical parameters—including French–American–British (FAB) classification, CALGB stage, and age (categorized as over 60 and under 60 years)—were conducted to further elucidate the model’s clinical relevance.

### 2.6 Immune microenvironment analysis

The association between high- and low-RS groups and the immune microenvironment was rigorously evaluated using two complementary bioinformatic approaches. Initially, the CIBERSORT algorithm (https://cibersortx.stanford.edu/) was employed to estimate the relative proportions of 22 distinct immune cell types in AML samples [15]. In parallel, the xCell algorithm (https://xcell.ucsf.edu/) was utilized to further quantify the relative abundances of immune cell populations between the RS groups [16]. The xCell signatures were validated through extensive in silico simulations and corroborated by cytometry-based immunophenotyping, thereby bolstering confidence in the accuracy of the immune profiling. Finally, heatmaps were generated using the ggcorr package to illustrate the correlation matrices among the immune scores derived from both CIBERSORT and xCell analyses

### 2.7 Somatic mutation analysis

To evaluate the interplay between the prognostic risk score (RS) and the mutational landscape in AML, masked somatic mutation (MAF) data from the TCGA-LAML cohort were retrieved from the Genomic Data Commons. Mutation profiles corresponding to simple nucleotide variations were extracted, and the frequency of the most prevalent mutations was determined. For comparative analysis between the high-RS and low-RS groups, the maftools package was employed. Importantly, to avoid bias from rare events, only genes mutated in at least five samples in one of the cohorts were considered.

### 2.8 Statistical analysis

All statistical analyses were performed in R software (version 4.4.2). For comparisons among multiple groups, the Kruskal–Wallis test was employed, whereas the Wilcoxon rank-sum test was used for pairwise comparisons. A significance threshold of p < 0.05 was applied throughout. This comprehensive statistical framework, which includes survival analysis, ROC curve analysis, and correlation assessments, ensures a robust evaluation of the prognostic model and its association with both clinical features and the immune microenvironment.

## 3 Results

### 3.1 Differential expression analysis between healthy individuals and AML patients

**Fig 1:**
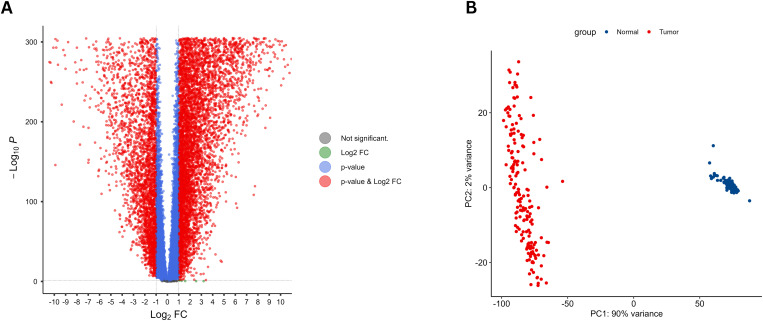
Differential expression analysis in AML. **(A)** Volcano plot illustrating the differentially expressed genes (DEGs) between AML samples and healthy bone marrow, with significant upregulation and downregulation clearly demarcated; **(B)** Principal component analysis (PCA) plot demonstrating distinct clustering of AML and healthy samples.

Differential expression analysis identified a total of 15,377 DEGs when comparing AML samples to healthy bone marrow. Among these, 11,202 genes were significantly upregulated and 4,175 were significantly downregulated in AML patients (Fig 1A). The PCA further confirmed that the expression profiles of AML and healthy samples were entirely distinct, as evidenced by the clear separation of clusters (Fig 1B). These 15,377 DEGs were subsequently designated as the diagnostic DEGs for further analyses.

### 3.2 Identification of diagnostic apoptosis-related DEGs

A comprehensive set of genes associated with cell death modalities—including apoptosis, pyroptosis, autophagy, necroptosis, and ferroptosis—was retrieved from the REACTOME database using the msigdbr package, yielding 537 unique genes (Supporting information 1). Intersecting this curated gene set with the diagnostic DEGs resulted in a subset of 451 genes, which were defined as diagnostic apoptosis-related DEGs (Supporting information 2).

### 3.3 Identification of prognostic and diagnostic apoptosis-related DEGs

Within the TCGA-LAML cohort, only patients with complete clinical and survival data (n = 130) were included for prognostic analysis. A univariate Cox regression analysis was performed on the diagnostic apoptosis-related DEGs, applying a log-rank cutoff of 0.05. This analysis identified 129 genes that were significantly associated with overall survival. These 129 genes were subsequently classified as prognostic and diagnostic apoptosis-related DEGs (PDA-DEGs) and served as the basis for the prognostic model.

### 3.4 Construction of the prognostic model

**Fig 2:**
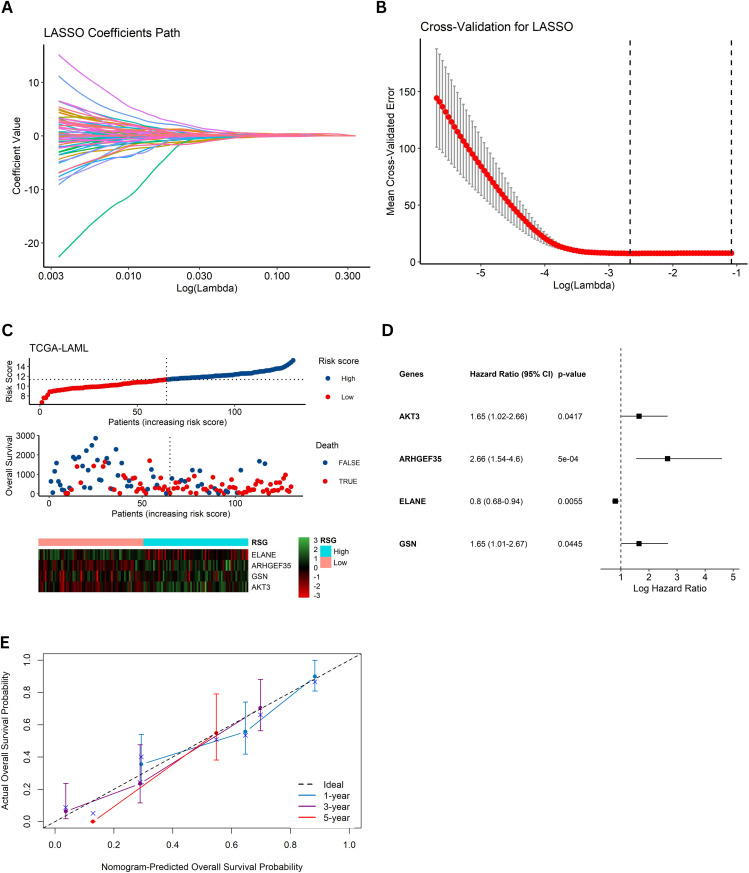
Construction of the prognostic model. **(A)** LASSO regression curve displaying the selection of candidate DEGs using 10-fold cross-validation in the training dataset; **(B)** Cross-validation plot indicating the optimal shrinkage parameter for model selection; **(C)** Multivariate Cox regression forest plot and heatmap of the four key genes (ARHGEF35, GSN, ELANE, and AKT3) that form the basis of the risk score (RS); **(D)** Forest plot summarizing hazard ratios for the candidate genes; **(E)** Concordance index (c-index) plot demonstrating the predictive accuracy of the RS model for 1-, 3-, and 5-year survival.

To construct a comprehensive multi‐gene prognostic model, the TCGA-LAML dataset was randomly partitioned into training (60%) and testing (40%) subsets. LASSO regression with 10-fold cross-validation was applied to the training set to reduce dimensionality and identify a panel of 21 DEGs (Figs 2A and 2B). These candidate genes were then incorporated into a multivariate Cox regression model, from which a RS was derived using a gold-standard formula: RS = (ARHGEF35 × 0.979157) + (GSN × 0.497877) + (ELANE × –0.223256) + (AKT3 × 0.499005). The expression patterns of these four key genes were further visualized in a heatmap comparing high- and low-RS groups (Fig 2C). Model performance was evaluated using forest plots (Fig 2D), and the concordance index (c-index) demonstrated promising predictive accuracy for 1-, 3-, and 5-year survival outcomes (Fig 2E).

### 3.5 Survival analysis and ROC evaluation

**Fig 3:**
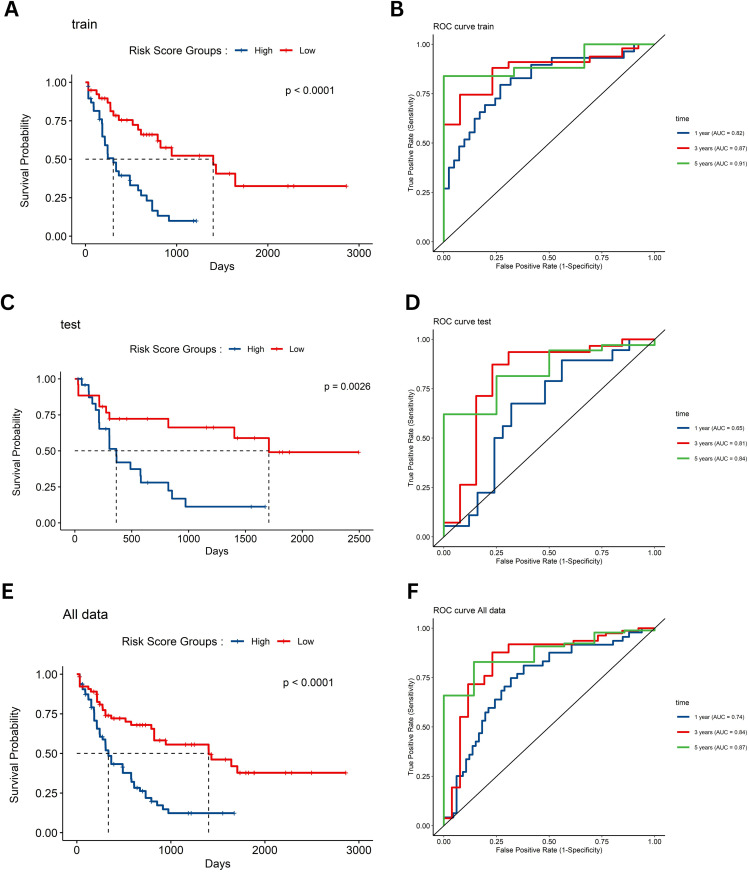
Survival analysis and ROC evaluation. **(A, C, and E)** Kaplan–Meier survival curves for the training, testing, and combined datasets, respectively, showing that increased RS is associated with significantly reduced overall survival; **(B, D, and F)** Receiver operating characteristic (ROC) curves for 1-, 3-, and 5-year survival in the training, testing, and combined datasets, respectively, which illustrate the robust predictive performance of the RS model.

Kaplan–Meier survival analyses were conducted on the training, testing, and combined datasets to assess the prognostic impact of the RS generated by our multi‐gene model. These analyses revealed that an increasing RS is significantly associated with reduced overall survival across all cohorts (Figs 3A, 3C, and 3E). Complementary ROC curve analyses for 1-, 3-, and 5-year survival further demonstrated robust predictive performance, as illustrated by the high AUC values observed in both the training and testing sets (Figs 3B, 3D, and 3F).

### 3.6 External validation of prognostic model

**Fig 4:**
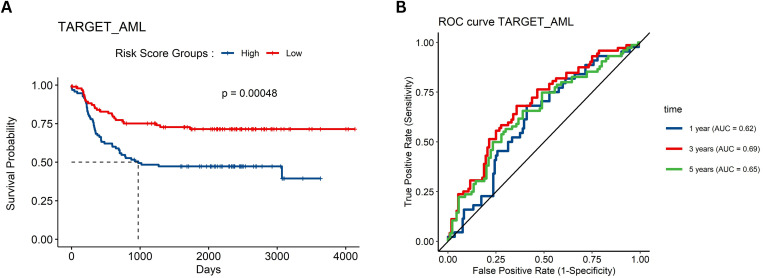
External validation of the prognostic model. **(A)** Kaplan–Meier survival curve from the TARGET-AML cohort, demonstrating that a high RS correlates with poorer overall survival; **(B)** ROC curves for 1-, 3-, and 5-year survival in the TARGET-AML cohort, confirming the model’s predictive capability in an independent patient dataset.

In the TARGET-AML cohort (n = 312), Kaplan–Meier survival analysis demonstrated that high RS was significantly associated with poorer overall survival (*p-value* < 0.00048; Fig 4A). Additionally, ROC analyses for 1-, 3-, and 5-year survival in the TARGET dataset yielded AUC values of 62, 69, and 65, respectively (Fig 4B).

### 3.7 Nomogram construction and clinical correlation

**Fig 5:**
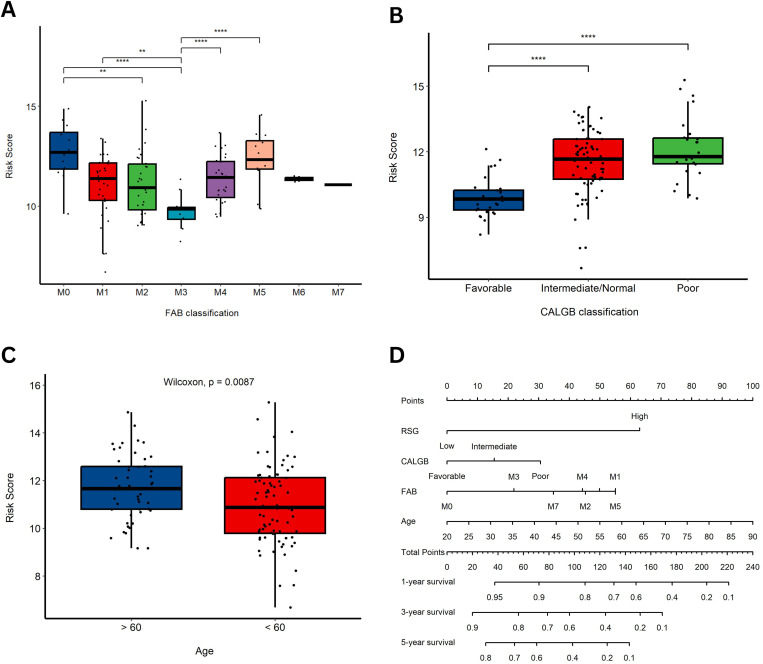
Nomogram construction and clinical correlation. **(A)** Boxplot depicting the distribution of RS across FAB subtypes; **(B)** Comparison of RS among different CALGB classifications; **(C)** Comparison of RS between patients older than 60 years and those younger than 60 years; **(D)** Nomogram that integrates RS with key clinical variables (age, FAB classification, and CALGB stage) to provide individualized 1-, 3-, and 5-year survival predictions.

A comprehensive analysis was performed to assess the clinical relevance of the RS by correlating it with established prognostic factors in AML. In our evaluation of FAB subtypes, the M3 subtype exhibited the lowest RS (Fig 5A). Analysis of CALGB classification revealed that patients with poor or intermediate/normal profiles had significantly higher RS compared to those with a favorable profile (*p-value* < 0.0001 for both comparisons; Fig 5B). Furthermore, a significant difference in RS was observed between patients older than 60 years and those younger than 60 years (*p-value* = 0.0087; Fig 5C). These findings were integrated into a nomogram that combined RS with key clinical parameters—age, FAB classification, and CALGB stage—to provide individualized survival predictions at 1, 3, and 5 years (Fig 5D).

### 3.7 Somatic mutation analysis

**Fig 6:**
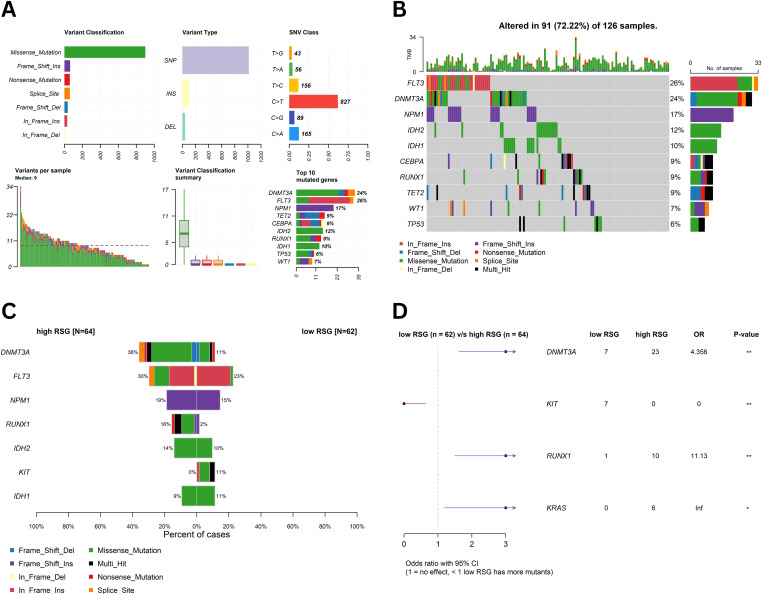
Mutation analysis in AML. **(A)** Summaries of variant classification, variant type, SNV class, and variants per sample in patients from the TCGA-LAML cohort; **(B)** Oncoplot indicating that 91 (72.22%) of the patients harbored at least one mutation in the top mutated genes; **(C)** Comparison of the most frequently mutated genes in high- (n = 64) versus low- (n = 62) RS groups; **(D)** Forest plot showing the odds ratios for selected gene mutations.

Somatic mutation profiles were examined in 126 AML patients from the TCGA-LAML cohort, of whom 91 (72.22%) harbored at least one mutation in the top ten most frequently mutated genes (DNMT3A, FLT3, NPM1, TET2, CEBPA, IDH1, IDH2, RUNX1, WT1, and TP53) (Figs 6A and 6B). Among these, DNMT3A (26%), FLT3 (24%), and NPM1 (23%) exhibited the highest mutation rates, followed by TET2 (12%), CEBPA (11%), IDH1 (10%), IDH2 (10%), RUNX1 (9%), WT1 (7%), and TP53 (6%). Variants per sample ranged widely, with some patients displaying more than 30 mutations. Moreover, missense mutations constituted the predominant variant classification, accompanied by frameshift insertions, nonsense mutations, and other less frequent events.

Subsequently, patients were stratified into high- and low-RS groups, and genes mutated in at least five samples in one of the groups were evaluated for significant differences (Fig 6C). Notably, DNMT3A (*p-value* = 0.0015) and RUNX1 (*p-value* = 0.0086) emerged as significantly enriched in the high-RS group, whereas KIT (*p-value* = 0.0058) was significantly more prevalent in the low-RS group (Fig 6D). Other frequently mutated genes, including FLT3, IDH1, IDH2, TET2, NPM1, and TP53, did not display statistically significant differences between the two RS groups after multiple testing correction.

### 3.8 Immune microenvironment analysis via CIBERSORT and xCell

**Fig 7:**
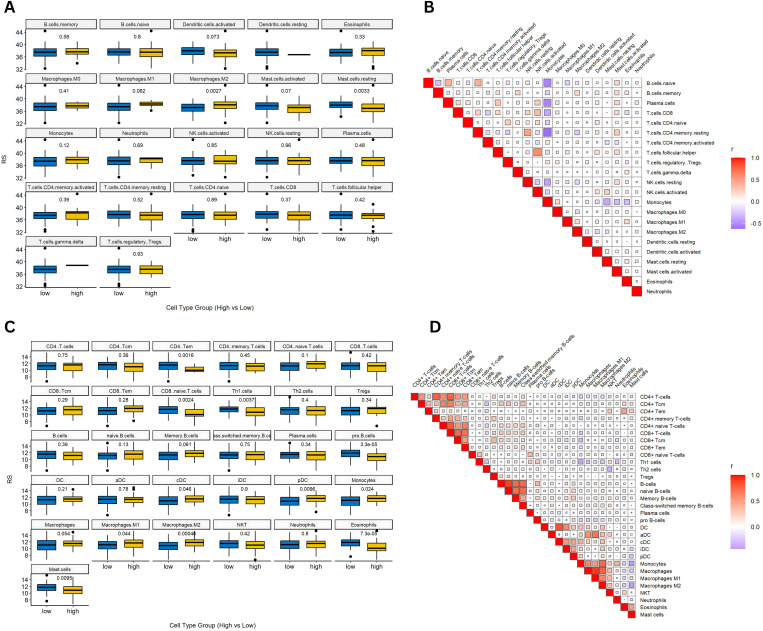
Tumor immune microenvironment analysis using CIBERSORT and xCell. **(A)** Bar plot of estimated relative proportions of 22 immune cell types in AML samples obtained by CIBERSORT, stratified by RS groups; **(B)** Heatmap of immune scores derived from CIBERSORT, illustrating the correlation matrix among various immune cell populations; **(C)** Comparative immune cell composition analysis from the xCell algorithm between high- and low-RS groups; **(D)** Heatmap of immune scores obtained via xCell, revealing interrelationships among immune cell types within the tumor microenvironment.

We investigated the association between the RS and the immune microenvironment by employing both CIBERSORT and xCell analyses. Using CIBERSORT, we estimated the relative abundances of 22 distinct immune cell types within AML samples and stratified patients into high- and low-RS groups. Statistical comparisons revealed that the high-RS group exhibited a significantly elevated proportion of M2 macrophages (*p-value* = 0.0027) and a markedly reduced fraction of resting mast cells (*p-value* = 0.0033) (Figs 7A and 7B). Complementary xCell analysis provided additional insights, identifying statistically significant differences in several immune cell populations between the risk groups: CD4 + T effector memory (*p-value* = 0.0016), CD8 + naive T-cells (*p-value* = 0.0024), Th1 cells (*p-value* = 0.0037), pro B-cells (*p-value* = 3.3e-05), conventional dendritic cells (cDC, *p-value* = 0.046), plasmacytoid dendritic cells (pDC, *p-value* = 0.0096), monocytes (*p-value* = 0.024), macrophages M1 (p = 0.044), macrophages M2 (*p-value* = 0.00046), eosinophils (*p-value* = 7.3e-05), and mast cells (*p-value* = 0.0095) (Figs 7C and 7D).

## 4 Discussion

AML remains one of the most aggressive hematologic malignancies, marked by rapid proliferation of malignant myeloid progenitors that supplant normal hematopoietic cells, leading to severe cytopenias and immunosuppression [17]. Despite significant advances in chemotherapy and targeted therapeutics, overall survival in AML continues to be dismal and relapse rates remain unacceptably high, a testament to the disease’s intrinsic heterogeneity and the complex interplay of genetic and molecular aberrations [18,19]. This clinical challenge underscores the urgent need for robust prognostic biomarkers capable of precisely predicting clinical outcomes and informing personalized therapeutic strategies for diverse patient cohorts.

Initially, we conducted differential expression analyses comparing AML samples with healthy bone marrow, which yielded a comprehensive set of diagnostic DEGs. By intersecting these DEGs with curated gene sets associated with apoptosis, pyroptosis, autophagy, necroptosis, and ferroptosis, we identified a focused subset of RCD-related genes. Subsequent univariate Cox regression analysis in the TCGA-LAML cohort allowed us to isolate prognostic markers significantly correlated with overall survival. Employing LASSO regression to mitigate overfitting, we developed a multi-gene prognostic model that generated a RS for each patient, effectively stratifying them into distinct prognostic groups with markedly different clinical outcomes. This prognostic efficacy was validated internally via Kaplan–Meier survival curves and ROC analyses for 1-, 3-, and 5-year survival, and externally in an independent TARGET-AML cohort. Moreover, a nomogram integrating the RS with key clinical variables—such as age, FAB classification, and CALGB stage—was constructed to further refine its predictive precision. Importantly, the RS constructed in our study is based on the expression profiles of four key genes—ARHGEF35, GSN, ELANE, and AKT3—which collectively provide a robust framework for elucidating the mechanistic underpinnings of RCD dysregulation in AML and pave the way for subsequent in-depth analyses of their individual contributions.

AKT3 is a pivotal isoform within the PI3K–AKT–mTOR pathway, orchestrating crucial cellular processes such as proliferation, survival, and metabolism, which are particularly relevant in the context of AML [20,21]. Aberrant activation of AKT3 is implicated in the inhibition of mitochondrial apoptosis, where it shifts the balance towards anti-apoptotic protein expression and suppresses caspase activation, thus facilitating chemoresistance [21–23]. The posttranscriptional regulation of AKT3 by noncoding RNAs also adds a layer of complexity; for instance, the circular RNA circMYC acts as a sponge for miR-516a-5p, resulting in increased AKT3 expression and enhanced mitochondrial respiration and cell viability, while suppression of AKT3 via the miR-497-5p axis has been shown to induce apoptosis [24,25]. Clinically, elevated AKT3 levels have emerged as a robust prognostic indicator in AML, correlating with aggressive disease behavior, poor overall survival, and increased relapse rates [21,26]. Our study further corroborates these findings, demonstrating that AML patients with high AKT3 expression exhibit significantly worse outcomes compared to those with lower levels, underscoring its potential as both a prognostic biomarker and a therapeutic target. Inhibition of AKT3, either through small molecule inhibitors or microRNA modulation, has shown promise in preclinical models by restoring apoptotic pathways and enhancing sensitivity to chemotherapy [27–29].

Gelsolin (GSN) is a multifunctional actin-binding protein that plays a pivotal role in regulating cytoskeletal dynamics, cell motility, and apoptosis, thereby exerting significant influence on the progression and prognosis of various cancers [30–33]. As both an intracellular (cGSN) and extracellular (plasma GSN or pGSN) isoform exist, its regulation is complex and context-dependent, with its activity being modulated by factors such as calcium and phosphatidylinositol 4,5-bisphosphate (PIP2). In gastric carcinoma, for instance, Hao et al. (2024) demonstrated that low GSN expression is associated with shorter overall survival and poorer clinical outcomes, suggesting that GSN acts as a putative tumor suppressor in this context by potentially modulating key signaling pathways such as the c-erbB/EGFR and PI3K/Akt cascades [34]. Similarly, Yang et al. have identified GSN as a key prognostic hub in bladder cancer, where its expression correlates with disease progression and serves as a central mediator in gene co-expression networks that govern tumor aggressiveness [33]. In the context of hematologic malignancies, particularly AML, emerging evidence from Wątek et al. underscores that hypogelsolinemia—a marked reduction in plasma gelsolin levels—is observed in AML patients, especially during the early stages of sepsis, suggesting that decreased GSN may contribute to the dysregulated apoptotic and inflammatory responses characteristic of this disease state [31]. Moreover, the role of GSN in modulating sphingolipid signaling, as evidenced by its capacity to act as a scavenger for bioactive lipids like sphingosine-1-phosphate (S1P) and ceramide, links it directly to the regulation of apoptotic pathways and cell survival in leukemic cells [31]. In addition, the work by Han et al. indicates that pGSN plays an important role in the final stages of erythropoiesis by promoting the maturation and enucleation of erythroid cells, which may have further implications in myelodysplastic syndromes and AML where ineffective hematopoiesis is a major clinical problem [30]. Complementary findings from Aghamaleki et al. using artificial neural networks further support the diagnostic potential of GSN in chronic lymphocytic leukemia [32].

ELANE encodes neutrophil elastase, a serine protease localized in azurophilic granules that has emerged as a critical mediator of selective anticancer activity [35]. It exerts its tumoricidal effects by liberating the CD95 death domain, which subsequently interacts with histone H1 isoforms to trigger cancer cell death via mechanisms reminiscent of pyroptosis [36–39]. Importantly, ELANE selectively targets malignant cells while sparing normal cells—a specificity that is attributed to the elevated expression of CD95 and histone H1 isoforms in cancerous tissues [35]. Additionally, ELANE has been shown to upregulate γ-H2AX, a robust biomarker of DNA damage, thereby linking its activity to the induction of mitochondrial dysfunction and further amplification of apoptotic signaling in tumor cells [35]. In vivo studies have demonstrated that ELANE not only attenuates primary tumor growth but also inhibits distant metastasis through an abscopal effect mediated by CD8+ T cells, underscoring its significant immunomodulatory properties [36]. In the context of hematologic malignancies, our study revealed that ELANE expression is significantly elevated in AML samples relative to healthy controls and correlates with poorer overall survival, thereby supporting its role as a prognostic biomarker in leukemia [40]. Furthermore, evidence from lung cancer models indicates that ELANE promotes M2 macrophage polarization via PTEN downregulation, thus fostering a tumor-promoting microenvironment that enhances proliferation, migration, and invasion [41]. Consistently, our immune profiling analyses using both CIBERSORT and xCell demonstrated that high-risk AML patients exhibit a higher proportion of M2 macrophages, further reinforcing the clinical relevance of ELANE in modulating the tumor microenvironment.

ARHGEF35 is a novel Rho guanine nucleotide exchange factor (GEF) encoded by a protein-coding gene located on chromosome 7q35 [42]. As a RhoGEF, ARHGEF35 is postulated to activate Rho GTPases by facilitating the exchange of GDP for GTP, thereby orchestrating critical cellular processes such as cytoskeletal reorganization, cell migration, and signal transduction—mechanisms that are integral to regulated cell death (RCD) pathways and oncogenic transformation [43,44]. Given the nascent state of research on ARHGEF35, its precise molecular functions and prognostic impact in AML and other cancers remain incompletely defined, warranting further investigation. In our study, high-risk score groups exhibited significantly elevated expression of ARHGEF35, suggesting that its upregulation may contribute to the aggressive phenotype observed in these patients.

Our mutation analysis revealed that high-risk AML patients are enriched for mutations in key regulators such as DNMT3A and RUNX1, indicating that genetic and epigenetic disruptions are central to leukemic progression [45–47]. DNMT3A, a key de novo DNA methyltransferase responsible for establishing and maintaining methylation patterns at CpG dinucleotides, is mutated in approximately 20–22% of AML cases. These mutations, often occurring in its catalytic domain, result in aberrant methylation patterns that silence tumor suppressor genes while activating oncogenic pathways, thereby promoting clonal dominance and poor prognosis [48]. Similarly, RUNX1—a master transcription factor crucial for hematopoietic stem cell differentiation and lineage commitment—is mutated in 5–10% of AML cases. Loss-of-function alterations in RUNX1 disrupt the balance between self-renewal and differentiation, leading to impaired hematopoiesis and exacerbating leukemogenesis, which correlates with adverse clinical outcomes such as lower remission rates and shortened overall survival [47,49]. In contrast, KIT mutations—affecting a receptor tyrosine kinase essential for cell proliferation, survival, and differentiation—were predominantly observed in low-risk patients. These mutations are frequently associated with core-binding factor (CBF) AML, a subtype known for retaining differentiation capacity and exhibiting a relatively favorable clinical course, partly due to responsiveness to tyrosine kinase inhibitors [50,51]. This molecular dichotomy, when integrated with our RCD-based gene expression signature, reinforces the prognostic value of our multi-gene risk model and highlights distinct molecular pathways that may serve as promising targets for tailored therapeutic interventions in AML.

Despite the promising results, our study has several limitations that suggest important avenues for future research. First, the retrospective design may introduce inherent biases in patient selection and data quality, emphasizing the need for prospective clinical trials to validate our prognostic model. Second, while our immune profiling using bulk RNA-sequencing data with CIBERSORT and xCell provided valuable insights into immune cell composition, these methods lack the spatial resolution and dynamic insights offered by single-cell or spatial transcriptomics, which could further elucidate the tumor microenvironment’s role in AML. Third, our mutation analysis was limited by reliance on publicly available TCGA data; integrating more comprehensive genomic data, such as whole-genome or targeted sequencing panels, could enhance the resolution of mutational landscapes and better delineate their relationship with the risk score. Moreover, potential confounding factors related to heterogeneous treatment regimens were not fully accounted for, underscoring the importance of incorporating detailed therapeutic histories in future studies. Finally, although our multi-layered model integrates gene expression, mutational, and immune profiling data, further refinement by incorporating additional omics layers—such as epigenetic modifications and proteomic profiles—could yield even more precise prognostic tools. Future mechanistic studies using approaches like CRISPR-Cas9 gene editing or RNA interference to dissect the functional roles of key genes may also uncover novel therapeutic targets to overcome chemoresistance in AML.

## Conclusion

Our study underscores the critical prognostic relevance of dysregulated RCD pathways in AML, illuminating the complex interplay between aberrant cell death mechanisms, genetic mutations, and the immune microenvironment. These insights not only deepen our understanding of AML pathobiology but also open new avenues for the development of targeted therapies aimed at modulating these interdependent pathways. By integrating our multi-gene risk model with established prognostic frameworks, such as the European Leukemia Net (ELN) criteria, we anticipate a significant enhancement in risk stratification precision, ultimately guiding the design of clinical trials that focus on RCD modulation in specific AML subgroups. This integrative approach holds promise for advancing personalized medicine strategies that improve therapeutic outcomes and overall survival in patients with this challenging malignancy.

## Supporting information

S1 Table Curated gene set of 537 unique genes associated with regulated cell death (RCD) pathways retrieved from the REACTOME database. (CSV)

S2 Table Subset of 451 diagnostic apoptosis-related differentially expressed genes (DEGs) derived from the intersection of RCD-associated genes (Supporting Information 1) and DEGs identified between AML and healthy bone marrow samples. (CSV)
